# Predictors of Length of Stay in Intensive Care Unit After Coronary Artery Bypass Grafting: Development a Risk Scoring System

**DOI:** 10.21470/1678-9741-2019-0405

**Published:** 2021

**Authors:** Maryam Zarrizi, Ezzat Paryad, Atefeh Ghanbari Khanghah, Ehsan Kazemnezhad Leili, Hamed Faghani

**Affiliations:** 1 Critical Care Nursing, Dr. Heshmat Hospital, Guilan University of Medical Sciences, Rasht, Iran.; 2 Department of Nursing (Medical-surgical), GI Cancer Screening and Prevention Research Center (GCSPRC), School of Nursing and Midwifery, Guilan University of Medical Sciences, Rasht, Iran.; 3 Department of Nursing (Medical-surgical), Social Determinants of Health Research Center (SDHRC), School of Nursing and Midwifery, Guilan University of Medical Sciences, Rasht, Iran.; 4 Department of Biostatistics, Social Determinants of Health Research Center (SDHRC), Guilan University of Medical Sciences, Rasht, Iran

**Keywords:** Atrial Fibrillation, Chest Tubes, Checklist, Length of Stay, Coronary Artery Bypass, Intensive Care Units, Pulmonary Atelectasis, Records

## Abstract

**Introduction:**

To determine predictors of length of stay (LOS) in the intensive care unit (ICU) after coronary artery bypass grafting (CABG) and to develop a risk scoring system were the objectives of this study.

**Methods:**

In this retrospective study, 1202 patients' medical records after CABG were evaluated by a research-made checklist. Tarone-Ware test was used to determine the predictors of patients' LOS in the ICU. Cox regression model was used to determine the risk factors and risk ratios associated with ICU LOS.

**Results:**

The mean ICU LOS after CABG was 55.27±17.33 hours. Cox regression model showed that having more than two chest tubes (95% confidence interval [CI] 1.005-1.287, Relative Risk [RR]=1.138), occurrence of atelectasis (95% CI 1.000-3.007, RR=1.734), and occurrence of atrial fibrillation after CABG (95% CI 1.428-2.424, RR=1.861) were risk factors associated with longer ICU LOS. The discrimination power of this set of predictors was demonstrated with an area under the receiver operating characteristic curve and it was 0.69. A simple risk scoring system was developed based on three identified predictors that can raise ICU LOS.

**Conclusion:**

The simple risk scoring system developed based on three identified predictors can help to plan more accurately a patient's LOS in hospital for CABG and can be useful in managing human and financial resources.

**Table:** 

Abbreviations, acronyms & symbols				
AF	= Atrial fibrillation		Exp(B)	= Exponentiation of the B coefficient
AUC	= Area under the curve		IABP	= Intra-aortic balloon pump
CABG	= Coronary artery bypass grafting		ICU	= Intensive care unit
CI	= Confidence interval		LOS	= Length of stay
CPB	= Cardiopulmonary bypass		PEEP	= Positive End-Expiratory Pressure
df	= Degrees of freedom		ROC	= Receiver operating characteristic
Euro SCORE	= European System for Cardiac Operative Risk Evaluation		RR	= Relative Risk
			SE	= Standard Error

## INTRODUCTION

Coronary artery disease is the most common cause of death in the world, and it is estimated that about one-third of all deaths are due to this disease. The methods of treatment of coronary artery disease vary according to the disease’s conditions. Lifestyle changes, medications use, and medical or surgical procedures are many ways to treat it. Surgery is used in patients who fail to control it with medical treatments or lifestyle changes^[1]^. The coronary artery bypass grafting (CABG) is the most common treatment option and a main treatment approach aiming to improve quality of life and reduce cardiac-related mortality^[2,3]^.

Despite the beneficial effects of CABG in controlling the signs and symptoms of coronary artery disease^[2]^, this surgery has many short and long-term complications. Because of these complications, all patients after CABG must be treated in the intensive care unit (ICU)^[4,5]^. The length of stay (LOS) of a patient after CABG in the ICU depends on the patient's individual characteristics, pre- and intraoperative care, complications, and events during surgery, as well as hospital policies and regulations. In many cardiac surgery centers, patients stay in the ICU for < 24 hours; in other centers, they stay up to 14 hours after CABG^[6,7]^. Prolonged ICU stay is an important predictor of adverse immediate, short-term, and long-term outcomes after cardiac operations and may be associated with increased hospital mortality rates^[4]^. This means that although caring for patients after cardiac surgery in ICU will help them to recover faster, prolonged ICU LOS can also present complications^[8,9]^. Therefore, determining the best time to be discharged from ICU after cardiac surgery can reduce the complications of prolonged stay in this setting^[10,11]^.

In addition, many cardiac surgery centers have a limited number of beds in post-cardiac ICU. Increasing bed occupancy time in the ICU increases the waiting time for other patients to have cardiac surgery. Determining factors that can influence the ICU LOS after CABG can help cardiac center managers to plan accurately the referral of candidates for elective surgery. Therefore, the present study aimed to identify risk factors of long-term stay in the ICU after CABG. The findings of this research can help to manage human and financial resources in line with accurate planning.

## METHODS

### Study Design

This observational retrospective study investigated factors related to ICU LOS after CABG and a score model was designed based on these factors. In this study, medical records of patients who underwent CABG in a specialty cardiac hospital in Rasht, a city in northern Iran, were reviewed. For this purpose, all the medical records of patients who underwent CABG from April 1, 2015 to August 1, 2017 were reviewed, and 1386 CABG were done in this time range. The medical records of patients over 18 years of age, undergoing CABG without history of previous cardiac surgery and intra-aortic balloon pump (IABP) use, and who were admitted to ICU after surgery were included in the investigation. Patients who required heart valve surgery or structural repair of heart or underwent re-surgery during their stay in the ICU or needed cardiopulmonary resuscitation and reintubation were not included in the study. In the cases reviewed during this period, ten patients had died and ten patients have been subjected to IABP. Sixteen patients had concurrent CABG and valve replacement, 25 patients have returned to the operating room after being transferred to ICU, 35 patients underwent surgery without cardiopulmonary pump, and 88 medical records had incomplete information. Therefore, 1202 medical files that met the inclusion criteria were finally included in the study.

The tool applied for this research consisted of a researcher-made questionnaire, and its validity was determined by a qualitative content validity method. The questionnaires were given to ten members of the faculty of nursing, two cardiac surgeons, and master staff who were working in the ICU after heart surgery. The research tool was made up of three sections; the first section consisted of demographic characteristics. The second section contained ten terms related to the underlying and surgical diseases, drug allergies, history of smoking and narcotics, and the left ventricular ejection fraction. The third section consisted of three subsets of preoperative (four items), intraoperative (20 items), and postoperative factors (24 items). Finally, the ICU LOS was mentioned (hours).

Data collection was carried out according to the criteria for entering the research, after obtaining the ethics committee’s confirmation. The ethical code of the study is IR.GUMS.REC.1396.281. All patients' medical records, the nursing reports of ICU, and the pump and anesthetic sheets, which were archived in the hospital, were assessed. The files of patients who underwent CABG during April 1, 2015 to August 1, 2017 were assessed based on the inclusion criteria.

Their LOS was longer than the mean ICU LOS, and it was considered as a long-term stay. Descriptive statistics (mean, standard deviation, and median) were used to describe the data. Kolmogorov-Smirnov test showed that the data did not follow the normal distribution. Tarone-Ware test was used to determine the relationship between each variable and LOS in ICU. *P* < 0.05 was considered as the significant level. Cox regression model was used for investigating the effect of several variables upon the time of discharge from ICU after CABG. The receiver operating characteristic (ROC) curve was also used to determine the best cut-off point of the model for patients' LOS in ICU. Hosmer-Lemeshow test was used to measure the model goodness of fit. Data analysis was done with the SPSS Inc., released 2009 for Windows, version 18.0 (Chicago: SPSS Inc).

## RESULTS

The results showed that 68.5% of the patients were males and the rest were females. Their mean age was 60.34±8.77 (ranging from 31 to 87) years; 30.4% of them were smokers and 18.1% were drug users. The most common underlying disease was hypertension (73.9%) and a history of transient ischemic attack had the least frequency (1.3%). Cardiac dysrhythmias were present in 1.6% of the patients before the surgery and the left main coronary artery had varying degrees of obstruction in 19.6% percent of the patients. The mean left ventricular ejection fraction was 45.17±9.78 (minimum and maximum ejection fraction were 15% and 65%, respectively); 20.4% of the surgeries were performed on an emergency basis and the rest was elective. At the study center, patients with any degree of occlusion in left main coronary artery are urgently admitted and surgically treated. Other patients should be on the waiting list for surgery. The mean time of cardiopulmonary bypass and aorta clamp were 59.96±15.95 and 36.59±10.86 minutes, respectively. About the postoperative factors, the mean tracheal tube durability was 244±582.51 minutes. The mean drainage from chest tubes was 559.44±312.56 cc in ICU (minimum of 100 and maximum of 2350 cc). The mean duration of chest intubation was 51.44±13.44 hours; in 962 cases (80%), the duration of chest intubation was less than the mean duration time. The minimum and maximum LOS for the chest tubes were 33 and 168 hours, respectively. The distribution of this variable was asymmetric and skewed. The most common dysrhythmia experienced by patients after surgery was atrial fibrillation and it occurred in 12.1% of them. The most common respiratory complication was atelectasis. Research findings indicated that the mean ICU LOS was 55.27±17.33 hours (minimum and maximum of 33 and 192.6 hours, respectively). The LOS of 26.2% of patients was more than the mean ICU LOS. Kolmogorov-Smirnov test showed the ICU LOS did not have normal distribution.

The Tarone-Ware test was used to determine the relationship between ICU LOS after CABG and demographic and other variables of study (Table 1). Afterwards, all the variables that had their significant relationship with the ICU LOS determined were entered into the Cox regression model by using backward likelihood ratio method.

**Table 1:** Comparison of the length of stay in the intensive care unit (hours) after coronary artery bypass grafting.

Variables		n	Mean	Median	95% Confidence interval		Significance*
					Mean	Median	
Age (year)	≤ 60	615	53.75	47.50	52.56-54.94	47.16-47.83	0.008
	> 60	587	56.87	48.00	55.30-58.43	47.66-48.33	
Creatinine (mg/dl)	≤ 1.2	929	54.67	47.60	53.55-55.79	47.37-47.82	0.005
	> 1.2	273	57.33	48.00	55.32-59.35	47.45-48.54	
CPB (min)	≤ 60	647	54.27	47.50	53.00-55.54	47.25-47.74	0.016
	> 60	555	65.44	48.00	54.93-57.96	47.72-48.27	
Intubation time (h)	≤ 6	6	48.55	44.00	40.42-56.69	42.68-45.32	0.003
	> 6	1196	55.31	47.75	54.32-56.29	47.57-47.92	
T-tube time	Yes	833	55.61	48.00	54.52-56.70	47.73-48.26	0.0001
	No	369	54.52	47.00	52.49-56.55	46.68-47.31	
PEEP	Yes	1140	54.98	47.60	53.99-55.98	47.41-47.78	0.028
	No	62	60.58	48.50	55.54-65.61	45.99-51.00	
Dysrhythmias	No	1057	53.31	47.50	52.41-54.21	47.31-47.69	0.0001
	AF	72	78.48	71.60	72.16-84.80	69.98-73.21	
	other	73	60.79	48.25	56.40-65.19	43.48-53.01	
Inotropic drugs	Yes	270	59.36	48.25	56.69-62.04	47.66-48.83	0.001
	No	932	54.09	47.50	53.10-55.08	47.31-47.69	
Drainage (cc)	≤ 560	765	53.17	47.50	52.15-54.19	47.29-47.71	0.0001
	> 560	437	58.95	48.20	56.99-60.92	47.79-48.60	
Pack cell	Yes	1011	56.08	47.90	54.97-57.19	47.68-48.11	0.0001
	No	191	50.99	47.00	49.26-52.73	46.42-47.57	
Chest tube	2	650	53.51	47.00	52.25-54.77	46.74-47.26	0.0001
	3	510	57.31	48.00	55.71-58.91	47.67-48.32	
	4	42	57.80	49.00	52.99-62.61	48.31-49.68	
Atelectasis	Yes	15	101.30	94.80	80.235-122.365	86.91-102.69	0.0001
	No	1187	54.69	47.70	53.78-55.60	47.51-47.88	
Hemothorax	Yes	9	93.06	73.50	61.85-124.26	66.19-80.80	0.001
	No	1193	54.99	47.70	54.04-55.93	47.51-47.88	
Pneumothorax	Yes	7	97.25	97.50	82.33-112.18	78.89-116.10	0.0001
	No	1195	55.03	47.70	54.04-55.99	47.51-47.88	
Duration of chest tube	≤ 51.5	962	49.36	47.00	48.68-50.04	46.84-47.15	0.0001
	> 51.5	240	78.97	71.60	76.64-81.31	71.19-72.00	
Bed sore	Yes	154	60.59	48.40	56.91-64.27	47.79-49.00	0.001
	No	1048	54.49	47.60	53.51-55.47	47.40-47.79	
History of diabetes	Yes	526	54.98	47.70	53.57- 56.38	47.41-47.98	0.76
	No	676	55.50	47.75	54.14-56.86	47.44-47.95	

AF=atrial fibrillation; CPB=cardiopulmonary bypass; PEEP=Positive End-Expiratory Pressure

* Tarone ware

**Fig. 1:**
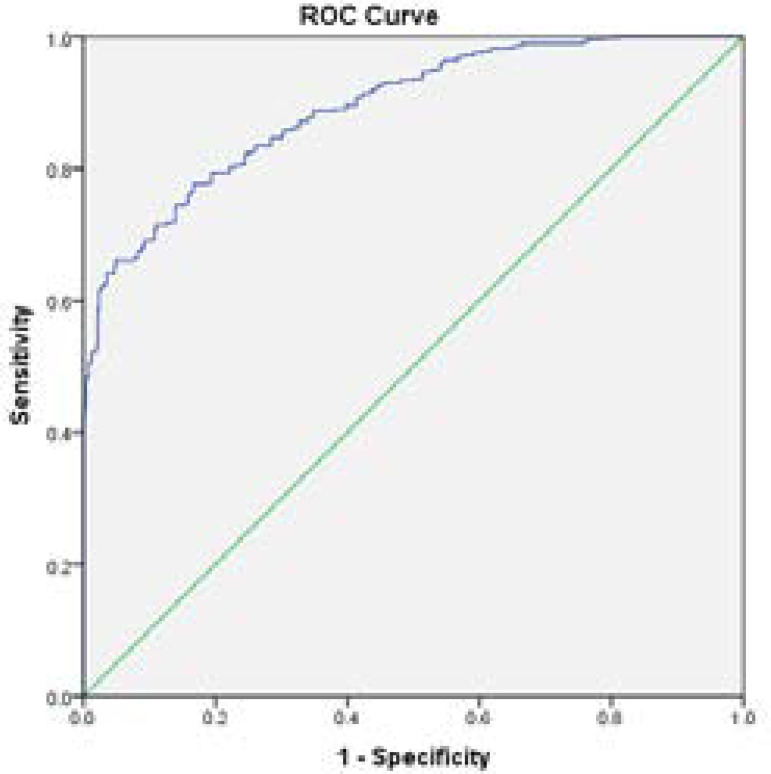
Receiver operating characteristic (ROC) prediction model for longer stay than 55 hours (area under the curve=0.697).

According to the results, number of chest tubes (95% confidence interval [CI] 1.005-1.287, Relative Risk [RR]=1.138), occurrence of atelectasis (95% CI 1.000-3.007, RR=1.734), and occurrence of atrial fibrillation (95% CI 1.428-2.424, RR=1.861) were identified as risk factors associated with longer stay in ICU after CABG (Table 2). Based on these findings, three predictors made a statistically significant contribution to ICU LOS after CABG. A non-significant Hosmer-Lemeshow test demonstrated that this set of predictors adequately predict ICU LOS after CABG. The discrimination power of this set of predictors was demonstrated with an area under the ROC curve (Figure 1); and the area under the curve was also measured. Based on these results, the area under the curve was 0.697 for the 55-hour LOS model (95% CI 0.663-0.731, *P*=0.0001) (Table 3). The respective weightings of the predictors were 24.1 (number of chest tubes) (> 2 chest tubes), 36.6 (occurrence of atelectasis), and 39.3 (occurrence of atrial fibrillation). One’s cumulative risk is determined by the summation of the weightings of the respective predictors, which ranges from 0 to 100. Higher scores indicate greater risk of longer stay in ICU after CABG.

**Table 2:** Cox regression hazard model analysis for identifying potential risk factors for length of stay in intensive care unit.

Variable	B	SE	Wald	df	Significance*	Exp(B)	95.0% CI for Exp(B)
Number of chest tubes	0.129	0.063	4.173	1	0.041	1.138	1.005-1.287
Atelectasis	0.550	0.281	3.840	1	0.050	1.734	1.000-3.007
Dysrhythmia			22.329	2	0.0001		
Atrial fibrillation	0.621	0.135	21.174	1	0.0001	1.861	1.428-2.424
Other dysrhythmia	0.185	0.131	1.971	1	0.160	1.203	0.930-1.556

* Cox regression

CI=confidence interval; df=degrees of freedom; Exp(B)=exponentiation of the B coefficient; SE=Standard Error

**Table 3:** Area under the receiver operating characteristic curve of length of stay in intensive care unit after coronary artery bypass grafting.

95% Confidence interval		Significance	SE	AUC	Discharge time
Upper bound	Lower bound				
0.731	0.663	0.0001	0.017	0.697	55 hours

AUC=area under the curve; SE=Standard Error

## DISCUSSION

Our results indicated that the LOS in about one third of the patients in this study was longer than the mean ICU LOS. Results of a similar study in Iran showed that patients staying in ICU setting was 2.2±1.5 days after CABG^[12]^. Also, the results of a study on elderly patients undergoing cardiac surgery in Italy showed that their mean ICU LOS was 1.9±1.5 days^[13]^. The number of patients waiting for cardiac surgery may affect the ICU LOS. Sometimes, due to the large number of patients awaiting surgery, patients with a stabilized hemodynamic status are discharged from the ICU earlier. This may even affect hospital policies. Since all patients require critical care after cardiac surgery, this will shorten the waiting time for surgery. The differences in the findings of some studies^[2,7]^ may be due to the difference in the number of intensive care beds compared to patients referred for surgery. Relevant references do not specify the standard time for staying in intensive care after cardiac surgery; depending on the patient’s condition, the LOS in this ward is determined. Of course, the primary decision maker in this case is the surgeon. Certainly, some of the patients’ characteristics can extend their stay in critical setting. However, it is not possible to be discharged from the ICU until the critical phase after surgery is passed and the patient's condition is stabilized. Sometimes, differences in care practices may also affect length of staying in ICU after cardiac surgery^[14]^.

The findings of the present study indicated that the number of chest tubes is one of the factors associated with increased ICU LOS after cardiac surgery. Lee et al.^[15]^ found that there was no difference between placing a chest drainage tube after cardiac surgery and more than one in terms of postoperative complications and its consequences. Our finding is inconsistent with the results of that study. In that study, Lee examined patients who underwent heart valves surgery, but, in this study, only medical records of patients undergoing CABG were examined. Because of the use of the internal mammary artery in this operation, pleural effusion is more likely. This may be the reason for the differences in the findings of the two studies. Placing the chest secretion drainage tube after cardiac surgery is largely related to intraoperative events. Even in the case of early removal of chest tubes, if the drainage rate is < 150 ml in the last four hours, it will increase the risk of complications associated with the patient's effusion^[16]^. However, an increase in the number of chest tubes and persistence of secretion indicate pulmonary complications and may predict a longer ICU LOS after CABG. Patients undergoing CABG are usually admitted to the ICU with at least two tubes due to cardiac manipulation. When more than two chest tubes are inserted for these patients, it indicates pleural damage and greater discharge probability. Therefore, it is expected that chest tubes will be longer in thorax and affect the ICU LOS. The second predictor of ICU LOS was atelectasis. Atelectasis is the most common respiratory complication after cardiac surgery^[17]^. Respiratory complications after cardiac surgery are almost inevitable. Cardiopulmonary pump use during open heart surgery, inactivation of the lungs during use of the pump, and the prolonged use of the pump can aggravate respiratory complications^[18]^. The result of the study by Naveed et al.^[19]^ showed that the most common respiratory complication after cardiac surgery is atelectasis. Following atelectasis, part of the lungs does not participate in the breathing process. In addition, due to sternum incision pain, the patient's breathing is superficial. Atelectasis can cause the patient's respiratory insufficiency^[17]^. The complexity of these complications seems to be able to predict longer stay in ICU after CABG. Although the final model of this study did not identify age as a predictor, due to the fact that this surgery is usually performed in middle-aged and elderly people, a later recovery of respiratory complications is expected^[19]^, increased stay in the ICU after CABG would be predictable.

Occurrence of atrial fibrillation was the third predictor of ICU LOS after CABG. Results of several studies approved the relationship between ICU LOS after the cardiac surgery and the occurrence of atrial fibrillation^[20,21]^. Atrial fibrillation occurs in around 35% of cardiac surgery cases and has a peak incidence on postoperative day two^[22]^. In their study, Perrier et al.^[23]^ showed a prevalence of atrial fibrillation of 21%. D’Agostino et al.^[24]^ reported a 24.9% incidence of this complication after CABG. Most studies show that at least one in every five patients develops atrial fibrillation after CABG. According to the results of this study, the occurrence of atrial fibrillation could be one of the predictors of ICU LOS, and a more precise timing of ICU discharge can be planned for these patients.

Many predictive models have been developed for a prolonged ICU stay, such as Tribodaharat et al.^[25]^, Parsonnet^[26]^, and the European System for Cardiac Operative Risk Evaluation (EuroSCORE)^[27]^. Ettema, in his systematic review study, revealed that Parsonnet^[26]^ and EuroSCORE^[27]^ were the two most accurate predictor models for prolonged ICU stay after cardiac surgery^[28]^.

### Limitations

We made use of a data registry that includes all patients who underwent CABG and systematically recorded a large amount of information on preoperative, perioperative, and postoperative characteristics. A disadvantage of using registry data is that not all predictors of the models are available in the registry with exactly the same definition as used to develop our model. Many variables affecting the ICU LOS may not be included in this registration system. In some cases, discharge may be made sooner, due to the need for intensive care beds and the large number of patients on the waiting list despite the need for intensive care. These were among the limitations of the present study. That could not be controlled by retrospective studies. These limitations may be controlled by longitudinal studies.

## CONCLUSION

This model was specially developed for isolated CABG surgery patients; and it cannot be used for patients undergoing valvular replacement surgery or other cardiac surgery. This study showed the best discrimination (0.69) in the CABG surgery population, and given its limitations, it seems to be useful in predicting ICU LOS after surgery.

**Table:** 

Authors' roles & responsibilities	
MZ	Substantial contributions to the conception or design of the work; or the acquisition, analysis, or interpretation of data for the work; agreement to be accountable for all aspects of the work in ensuring that questions related to the accuracy or integrity of any part of the work are appropriately investigated and resolved; final approval of the version to be published
EP	Substantial contributions to the conception or design of the work; or the acquisition, analysis, or interpretation of data for the work; drafting the work or revising it critically for important intellectual content; agreement to be accountable for all aspects of the work in ensuring that questions related to the accuracy or integrity of any part of the work are appropriately investigated and resolved; final approval of the version to be published
AGK	Substantial contributions to the conception or design of the work; or the acquisition, analysis, or interpretation of data for the work; agreement to be accountable for all aspects of the work in ensuring that questions related to the accuracy or integrity of any part of the work are appropriately investigated and resolved; final approval of the version to be published
EKL	Substantial contributions to the conception or design of the work; or the acquisition, analysis, or interpretation of data for the work; agreement to be accountable for all aspects of the work in ensuring that questions related to the accuracy or integrity of any part of the work are appropriately investigated and resolved; final approval of the version to be published
HF	Substantial contributions to the conception or design of the work; or the acquisition, analysis, or interpretation of data for the work; agreement to be accountable for all aspects of the work in ensuring that questions related to the accuracy or integrity of any part of the work are appropriately investigated and resolved; final approval of the version to be published
